# The complete chloroplast genome sequence of three medicinal species; *Curcuma longa, Curcuma wenyujin,* and *Curcuma phaeocaulis* (Zingiberaceae)

**DOI:** 10.1080/23802359.2020.1768917

**Published:** 2021-04-08

**Authors:** Min-Kyeoung Kim, Woo Kyu Lee, Yoo Rae Choi, Jonghwan Kim, Ilhyun Kang, Juhye Kang

**Affiliations:** Herbal Medicine Research Division, National Institute of Food and Drug Safety Evaluation, Ministry of Food and Drug Safety, Cheongju-si, Republic of Korea

**Keywords:** Chloroplast, genome sequencing, *C. longa*, *C. wenyujin*, *C. phaeocaulis*

## Abstract

*Curcuma longa, C. wenyujin* and *C. phaeocaulis* are important herbal medicine which of rhizomatous herbaceous perennial plant of the family Zingiberaceae. This study generated a complete chloroplast genome sequence of three medicinal species were characterized by de novo assembly with whole genome sequencing data. The length of complete chloroplast genome were 162,180 bp (*C. longa*), 162,266 bp (*C. wenyujin*), and 162,133 bp (*C. phaeocaulis*), respectively, with four structures that were included in large single copy region (87,001 bp, 87,042 bp, and 87,013 bp), small single copy region (15,681 bp, 15,710 bp, and 15,622 bp), and duplicated inverted regions (29,749 bp, 29,757 bp and 29,749 bp of each). Based on phylogenetic trees, *C. longa, C. wenyujin,* and *C. phaeocaulis* were grouped by high bootstrap value with *Curcuma* species. This result approved that *C. longa, C. wenyujin* and *C. phaeocaulis* were comprised in *Alpinia and Wurfbainia*. Therefore, this chloroplast genome data firstly generated valuable genetic resource in discrimination of herbal materials, phylogeny and development DNA marker.

*Curcuma longa, C. wenyujin*, and *C. phaeocaulis* are perennial plants belonging to the family Zingeraceae. The genus *Curcuma* consists of about 50 species in Southeast Asia, of which more than 12 species are found in China (Wu and Larsen 2000), widely cultivated in the subtropical regions of the world (Li et al. 2011).

These plants part of rhizoma have commonly used as spice, natural food dye, and medicine (Curcumae Longae Rhizoma and Zeodariae Rhizoma), widely used in Asia (Sigrist et al. 2011). In Ayurveda medicine, it is used as a treatment for inflammatory conditions; and, in traditional medicine, it is used as stimulant, aspirant, carminative, cordeal, emenagogue, astringent, detergent, and diuretic effects (Sasikumar 2005; Remadevi et al. 2007; Jurenka 2009). In this study, we determined the complete chloroplast genome sequences of three medicinal species, *C. longa, C. wenyujin*, and *C. phaeocaulis.* These sequences will be a valuable genetic resource for further molecular study and phylogenetic analysis of *Curcuma* species with other species in the family Zingeraceae.

The plant sample of *C. longa*, *C. wenyujin*, and *C. phaeocaulis* were supplied from National Institute of Horticultural and Herbal Science in Korea (Eumseong-gun, Chungcheongbuk-do, 36°56’39.6”N 127°45’17.3”E 36.944327, 127.754793). A voucher specimen (*C. longa*; CUR16, *C. wenyujin;* CUR09, and *C. phaeocaulis*; CUR16) was deposited at the herbarium, in the National Center of Herbal Medicinal Resources at OK-chen (National Institute of Food and Drung Safety Evaluation, Korea).

The genomic DNA was extracted from dried leaf were stored at −70 °C refrigerator in the laboratory of the NIFDS (National Institute of Food and Drug Safety Evaluation), Herbal Medicine Research Division and assembled to an Illumina paired-end library. Genomic sequencing was performed using Illumina Hiseq 2000 instrument (Illumina, San Diego, CA) and assembled by CLC genomic assembler (v. beta 4.6, CLC Inc., Rarhus, Denmark).

The total size of the assembled chloroplast genome of *C. longa, C. wenyujin*, and *C. phaeocaulis* were 162,180 bp, 162,266 bp, and 162,133 bp, respectively (accession No. MK109018, MK109019, and MK109020), and genome reads were 215 X of average coverage. The three medicinal plant genome structure typically consisted of four parts that were large single copy region (LSC) of 87,001 bp, 87,042 bp, and 87,013 bp, small single copy region (SSC) of 15,681 bp, 15,710 bp, and 15,622 bp, and a pair of inverted regions (IRa and IRb) of 29,749 bp, 29,757 bp, and 29,749 bp each. In genome, a total 114 coding regions, which were comprised 80 protein-coding regions, four rRNA genes, and 30 tRNA genes, were predicted through OGDraw (http://ogdraw.mpimp-golm.mpg.de, Lohse et al. 2007) and manual curation using BLAST.

To determine the taxonomic status of *C. longa, C. wenyujin,* and *C. phaeocaulis*, phylogenetic analyses based on phylogenetic tree were performed from genome sequences of ten taxa, consisting of genus *Curcuma, Alpinia*, and *Wurfbainia*. These sequences were aligned using MAFFT (http://mafft.cbrc.jp/alignment/software/, Katoh and Standley 2013). The Phylogenetic relationship was constructed by eight species, and 1000 bootstrap replications in MEGA6 (Tamura et al. 2013). Phylogenetic analysis was conducted with chloroplast genome sequences of *Curcuma longa, C. wenyujin*, and *C. phaeocaulis* with eight species belonging to the family Zingeraceae (Figure 1). This chloroplast genome data could be supported to discriminate between three similar medicinal species for herbal medicine and adulterations, analyze the relation of closely related taxa, and study phylogeny and identification marker.

**Figure 1.**
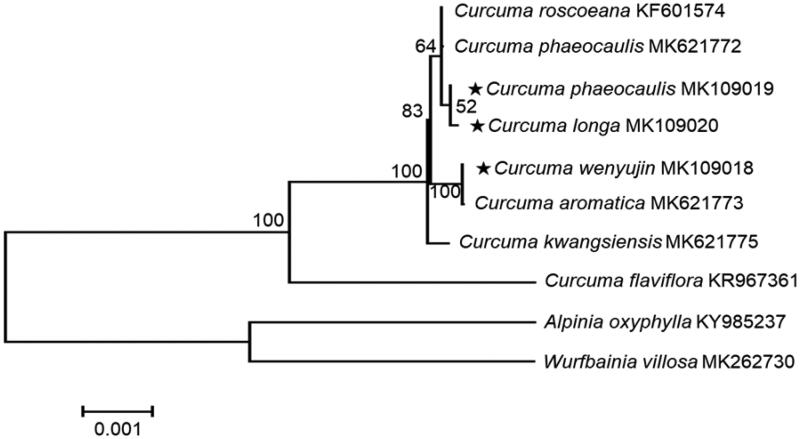
Maximum-likelihood analysis of *C. longa, C. wenyujin*, and *C. phaeocaulis* with related species in Zingiberaceae based on complete chloroplast genome sequences. Numbers on branch indicated bootstrap values.

## Data Availability

The data that support the findings of this study are openly available in NCBI, National Center for Biotechnology Information at https://www.ncbi.nlm.nih.gov/nuccore/MK109019.1/, https://www.ncbi.nlm.nih.gov/nuccore/MK109020.1/, and https://www.ncbi.nlm.nih.gov/nuccore/MK109018.1/, reference number [accession No. MK109018, MK109019, and MK109020].
